# Series: Public engagement with research. Part 3: Sharing power and building trust through partnering with communities in primary care research

**DOI:** 10.1080/13814788.2024.2328707

**Published:** 2024-03-28

**Authors:** Jessica Drinkwater, Michelle Farr, Gary Hickey, Esther Van Vliet, Sophie Söderholm Werkö, Ingrid Klingmann, Steven Blackburn

**Affiliations:** a Centre for Primary Care and Health Services Research, University of Manchester, United Kingdom; b The National Institute for Health and Care Research Applied Research Collaboration West (NIHR ARC West) at University Hospitals Bristol and Weston NHS Foundation Trust, Population Health Sciences, Bristol Medical School, University of Bristol, United Kingdom; c Agora Digital Centre, School of Healthcare Enterprise and Innovation, University of Southampton, United Kingdom; d Knowledge Transfer Office, Tilburg University, The Netherlands; e The Swedish Agency for Health Technology Assessment and Assessment of Social Services, Stockholm, Sweden; f European Forum for Good Clinical Practice (EFGCP), Brussels, Belgium; g Institute of Applied Health Research, University of Birmingham, United Kingdom

**Keywords:** Participatory research, public engagement, Co-production, power sharing

## Abstract

**Background:**

This article focuses on potential strategies to support primary care researchers in working in partnership with the public and healthcare professionals. Partnership working can potentially to improve the relevance and usefulness of research and ensure better research and health outcomes.

**Discussion:**

We describe what we mean by partnership working and the importance of reflecting on power and building trusting relationships. To share power in partnership working, it is essential to critically reflect on the multiple dimensions of power, their manifestations, and your own power. Power can influence relationships and therefore, it is essential to build trust with partners. Next, we outline how the context of primary care research and decisions about who you work with and how to work together, are vital considerations that are imbued with power. Lastly, we suggest different ways of working in partnership to address different dimensions of power. We provide examples from primary care research across Europe regarding how to recognise, tackle, and challenge, invisible, hidden and visible power.

**Conclusion:**

We conclude by proposing three calls to actions to encourage researchers working in primary care to consider the multiple dimensions of power and move towards partnership working. First is to use participatory methods to improve the inclusivity of your research. Second is to include patients and the public in decisions about the design, delivery and development of research and its outcomes. Third is to address various systemic and institutional barriers which hinder partnership working.

## KEY MESSAGES

Partnership approaches to primary care research can potentially improve the relevance, usefulness and inclusivity of research.

Working in partnership involves researchers and the public sharing power in important research decisions and building trusting relationships.

Recognising and addressing power differentials and building trusting relationships requires time and effort.

## Introduction

The importance of engaging patients, the public and/or communities in the design and delivery of health and social care research is recognised [1]. Not engaging with the public raises the risk that decisions about health and care are not fully informed by all the available evidence and perspectives, including patient knowledge and lived experience. The different approaches to how the public can be engaged in research and why you should engage them, are discussed in the first of this four-part series. These approaches vary in the extent to which power in decision-making is shared between public members, researchers and clinicians. This third article in the four-part series focuses on working in partnership with these different stakeholders to share power in decision-making in primary care research.

We explain what we mean by ‘partnership working’ and explore the multiple dimensions of power within a primary care research context. Whilst we acknowledge many systems of ‘power over’ us can constrain our actions, we can also tap into different forms of ‘power to’ achieve aims and ambitions and ‘power with’ others to achieve more than we could on our own [2]. Supported by case studies and helpful resources exploring various ways of sharing power and building trust, we identify different ways of working in partnership with people. Finally, we list calls to action to encourage primary care researchers to adopt a more collaborative research approach.

The authors include people with lived experience of primary health care services, public engagement professionals, clinicians, and researchers, all with an interest in and experience of partnership approaches to research. We hope the readers of this article have equally diverse backgrounds, and therefore, we will refer directly to ‘you’ rather than making assumptions about your academic background.

## What do we mean by partnership working in research?

There are many ways of doing research together with the public, including patients and other stakeholders [1]. In this article, we focus on working in partnership with people on research studies in which decision-making power is shared. Examples include co-production, co-creation, participatory research and citizen science, but other terms are also used. We acknowledge that there are both similarities and differences between these approaches, that they often overlap and there can be confusion around language and concepts [3].

In 1969, Arnstein created the ladder of citizen participation describing differences in how the public is involved in institutional decision-making based on their power and influence [4]. The ladder moves from approaches classified as non-participation (decisions are made about or for the public), through approaches which are tokenistic (the public are informed about a decision, or their views are collected for someone else to make the decision), to approaches where the public have direct power in decision-making. The higher rungs of the ladder cover partnership working and approaches where the public has increasingly more direct power and control in decision-making [4]. Importantly, although Arnstein’s analysis focused on the practice of sharing power, there was recognition that despite the rhetoric this is not always achieved [4] There have been critiques of Arnstein’s ladder [5], specifically that it focuses on a single dimension of power, when power is actually multi-faceted. The ladder focuses on decision-making around solutions rather than incorporating the public within problem formulation and needs to better account for the diversity of knowledge and experience of both health professionals and the public. Despite this it remains broadly relevant today and applicable to most institutional research processes. This article focuses on the commonalities of the different approaches to partnership working, which are striving to work in equal partnership or delegating decision-making power to people during the research process.

## The importance of reflecting on power and building trusting relationships

When contemplating partnership working, first, learn about power and critically reflect on your own power. Sharing power, developing trust and relationship building are vital skills in partnership working. Attempting to share power is highly challenging as different dimensions of power are embedded in everything we do [2]. Power can be visible within institutions, hierarchies, policies, economic resources, and rules; hidden through agenda-setting and opaque decision-making processes, or invisible within usual practice, language, beliefs, attitudes, values and assumptions, and what is considered knowledge [2,6]. These different dimensions of power are set out in Table 1.

**Table 1.** Dimensions of power [2] applied within a primary care research context (developed from [6,7]).

Dimensions of power	Description	Examples within the context of primary care research
Visible	Institutions, hierarchies, policies, economic resources, and rules	Professional hierarchies, where funding is located, contracts, rules and policies such as research governance processes
Hidden	Agenda-setting and opaque decision-making processes	How decisions about research agendas and public involvement are made
Invisible	Usual practice, language, beliefs, attitudes, values and assumptions	Preferred methodologies, forms of knowledge, how lived experiences are valued, usual medical practice, traditional patient-doctor relationships, culturally-based assumptions, unconscious bias

Visible power might include structural drivers of research such as publications, governance systems including ethical approval and data management, and payment for contributors, which may all constrain opportunities and alter priorities. Attention should also be paid to the institutional context in which the researchers are employed, which can often constrain what is possible in terms of partnership working [8]. Hidden dimensions of power can be associated with framing research problems and how decisions are made about what to research, why and with whom. Invisible aspects of power relate to our subconscious beliefs and cultural assumptions, which can be particularly difficult to spot as they can be unconscious. Thus critical reflective practice, and constant attention to fluctuating power dynamics are needed [9]. Being transparent about all these issues with non-academic partners is essential; researchers also need to challenge and change the systems that create inequities.

Secondly, prioritise building relationships and trust. This can help generate a sense of ‘power with’ where more becomes possible through partnership than achievable alone [2]. Respecting everyone’s humanity and diversity with care, compassion, creativity and humility is an essential starting point [10]. Developing relationships and trust between different stakeholders within partnership research takes time and requires emotional work [11]. This is true for all involved in working in partnership: patients and the public, researchers and health care professionals. Professionals may need to be more personable and expose their own vulnerabilities to support more equal relationships [12]. Trusting relationships develop over time through interactions, and this trust can be built or broken dependent on these interactions and events [11]. There are examples where co-design techniques and careful facilitation can help restructure relationships, acknowledging past hurts and emotional pain, to reconfigure relationships into more harmonious connections [13].

## Understanding the context of primary care research, who to partner with, and the influence of power

In Europe and worldwide, implicitly, most members of the public are also patients and registered users of primary care services. The huge scope of this primary care community is both an opportunity and a challenge for partnership working in primary care research. The opportunity is a large number of people to collaborate with. The challenge is defining who you are working with, why them and not someone else, and their power in society and within the research.

Primary care is at the interface of applying population-based evidence to individual patients, and there are times when there is tension between population interest and individual patient benefit. Thus, the public (people irrespective of whether they are receiving or registered for care) and patients (people who are receiving or registered to receive care) can occasionally have different or even opposite vested interests [14]. Vested interests implies that there may be visible, hidden, or invisible expectations of financial or other gain (such as health improvement). Potentially opposing vested interests may be especially apparent in publicly funded health systems, where decisions about new treatments or services may mean reallocating resources and withdrawing a different treatment or service. Decisions about resource allocation may be made to benefit the public (lower taxes, more efficient services, improved equity), but have a detrimental effect on individual patients. Therefore, it is essential to be explicit and transparent about who you are working with, what power they have in decision-making, their agenda about the research and discuss everyone’s interests, and how different perspectives can be accounted for.

As mentioned above, defining a community to work with is essential and complicated in primary care research. Community may be defined as a population within a specific geographic area or based on a particular characteristic of a population, such as disease, ethnicity, or similar lived experience (e.g. homelessness, addiction). Communities of interest are likely to be defined through project aims. For example, is the project exploring public health or individual patient care questions? Communities may be comprised of different stakeholders including individuals, campaign groups, voluntary, community, or social enterprise (VCSE) organisations and health and care organisations. We use the term stakeholder to refer to a person or group with a vested interest in decision-making about primary care. Working with an individual can make it easier to build a relationship; however, working with a VCSE organisation may mean that you have a broader and more diverse voice to collaborate with, alongside the VCSE’s expertise in working with specific groups. Partnership working may involve working with one or more different stakeholders. Some sensitive projects (such as those exploring historical power imbalances, people excluded from care, or people who have experienced abuse) may involve a single stakeholder group, whereas other projects will involve bringing different stakeholders together. Part 2 of this series on public engagement explores the importance of equity, diversity, and inclusion when working with different stakeholders [15]. It is essential to recognise, discuss and critically reflect with your potential stakeholders about everyone’s vested interests (including researchers) at the start and throughout any research project.

Your community may also include clinical and non-clinical staff employed by public, private, charitable or VCSE providers of primary care services. Any partnership bringing together an individual patient and doctor with a clinical therapeutic relationship needs to be managed with care [16]. Attention should be given to power and ethical collaboration issues, which may also affect the therapeutic relationship. However, multistakeholder partnership working emphasises reflection, which can lead to both patients and staff reflecting on their therapeutic relationships [17].

## Different ways of working in partnership to address invisible, hidden, and visible power

Everyone can work towards making primary care research more equitable, welcoming and inclusive, so that partnership working with communities can become more common. However, we accept that partnering with people and communities can initially feel daunting. Developing relationships and reflecting on power can be time-consuming and challenging. Equally there are institutional, cultural, and societal barriers to this work which are different depending on the country you work in.

In the next section, we outline three areas in which researchers can make a step change towards partnership working by addressing different power dynamics and increasing trust in relationships. First, we discuss how to reflect on and consider invisible power and make research more inclusive and equitable. Second, we illustrate how to share decision-making with the public about the research agenda and approach, challenging hidden power. Third, we explore addressing visible power by developing trusting relationships to open up institutions to communities. Where you focus your effort will depend on your own situation, the support and resources available, the power you have to effect change, and the research you are doing.

### Recognise invisible power and make research methods more inclusive and equitable

The first article in this series outlines how the public can be engaged throughout the research cycle irrespective of the methods used [1]. Invisible power is also present throughout the research cycle in terms of assumptions about what can be measured, unconscious bias within some research methods (e.g. relying on high literacy levels), culturally insensitivity, and the value given to different forms of knowledge resulting in epistemic injustice. Many participatory and co-design methods and tools specifically address invisible power and increase epistemic justice. There is a long tradition of participatory research methods originally developed through working with communities in the Global South [18]. These specific tools and techniques are creative and visual and aim to promote the voice of previously excluded communities. For example, using non-traditional forms of data (e.g. visual data in photovoice and other examples in case studies below [19]) can allow equity in the presentation of knowledge and priority setting even when there are language barriers or low literacy levels. These tools often include peer or community researcher data collection, which can be more culturally acceptable than an outsider coming into a community. They also aim to support practical research outcomes that can be directly used by local communities and, therefore, often have an immediate physical outcome such as a map or chart. One set of tools and techniques that have been used in primary care research is participatory learning and action (PLA) (case studies 1 and 2). These tools and techniques may address hidden power and make data generation more inclusive; however, they do not address hidden power within decisions about what is researched, with whom, and how it is researched, or visible power.

Case study 1. Participatory learning and action with marginalised groups

**Table:** 

*What happened* O’Donnell et al. [20] aimed to identify levers and barriers for marginalised groups accessing primary care in Ireland. They designed creative focus groups with stakeholders with diverse communication needs and abilities, including migrants, homeless people, travellers, and young mothers living in areas of deprivation. *Consequences* Within the focus groups they generated data using visual techniques to chart important themes experienced by those involved, as well as traditional audio recordings. This moves away from relying on spoken and written communication, which can be excluding for some marginalised groups. *Lessons* Visual techniques helped address the invisible power present in judgements about literacy and the ability of marginalised communities to contribute when the only option is through written or verbal communication.

Case study 2. Participatory learning and action bringing together diverse stakeholders

**Table:** 

*What happened* PLA tools were used in a Europe-wide study exploring the implementation of guidelines to enhance cross-cultural communication in primary care consultations with migrants [21]. The study involved migrants, interpreters, and primary care provider staff working together in partnership. *Consequences* The visual PLA tools helped develop a common communicating method across diverse stakeholders. *Lessons* The evaluation of the PLA techniques demonstrated that these techniques were experienced positively, created safety and trust in multi-stakeholder groups, helped to equalise power across different backgrounds and social status, and fostered learning across stakeholder groups.

### Tackle hidden power by sharing decision-making about the design and conduct of research

Partnership approaches to research take an explicit stance to work *with* communities and stakeholders affected by the research throughout the research and involve them in decision-making about the design and conduct of the research [22,23]. There are toolkits available to help support the use of participatory action research techniques [24] and resources to support the co-production of research [25]. Involving the public in research agenda-setting is a fundamental element of sharing power and researchers have used creative methods and facilitation to support these conversations [7,26]. In the UK the James Lind Alliance has developed approaches to support public involvement in research prioritisation, bringing together clinicians, patients and carers to identify and prioritise research questions [27]. Participatory research is signified by the involvement of stakeholders in designing the research question, interpreting the results, and agreeing on the dissemination messages [28]. These are all significant decision-making points. Collaborating in this way often takes longer and requires strong relationships of trust between researchers and stakeholders. In case studies 3 and 4, we outline examples of this type of research. Case study 3 involves researchers working with existing community groups; case study 4 involves researchers establishing specific groups of stakeholders to work on a project. Both examples demonstrate the need for long-term relationships, mechanisms for building trust, and developing power-sharing around significant decision-making.

Case study 3. Working with communities who experience severe health inequities

**Table:** 

*What happened* One participatory action research study worked with women with lived experiences of trauma to improve access to primary care services for marginalised groups. The project was initiated by a GP who worked at a community organisation. Her existing relationships with the VCSE organisation staff and the women with lived experience was a core catalyst for the collaborative development and planning of the project [16]. Women with lived experience, VCSE staff and GPs worked together in fortnightly meetings with researchers who facilitated the meetings. Having such regular meetings, this meant that everyone could be more involved in decision-making throughout the project. *Consequences* The group collaborated with different GP practices, including helping to co-design and implement a new specialised clinic and improved access to general practices for people who experience complex health needs.[29] An additional researcher who had no prior experience with the group conducted interviews with all involved to find out about their experiences of working together on service improvements. *Lessons* Crucial to the success of this project has been involving everyone in decision-making through regular meetings and trusting relationships. A VCSE support staff member attended all the fortnightly meetings, which was important to provide emotional and practical support for those with lived experience, as they already had a trusting relationship. The group found that it was essential to move away from sharing lived experiences, which could retrigger trauma. Researchers needed to understand trauma and how this may impact upon an individual’s sense of psychological safety. Making changes was the women’s primary motivation for getting involved. There was a huge sense of empowerment that came from seeing tangible change from combined efforts. Using participatory action research, they documented their learning about how they worked together, including creating safe spaces, sharing decision-making, and having an inclusive approach to working together [16].

Case study 4. Co-designing services with patient advisory groups and councils

**Table:** 

*What happened* A study by Haesebaert et al. established a new type of patient advisory council for primary care clinics which included patients [30], caregivers, clinicians and managers. The idea was that patients would help to co-design new services and improve service quality within the primary care clinics. The academic researchers worked closely with patient experts (patients trained in research and with existing relationships with the researchers) to design the study, recruit practices to participate and facilitate meetings. *Consequences* The patient experts acted as facilitators supporting the newly established patient and staff councils to work together. Patient experts, taking on the important role of facilitators within the research, helped to foster mutual respect between patient and staff council members. The councils helped produce practical proposals to improve patient experiences and facilitate patient-centred approaches. *Lessons* The authors highlight the importance of underlying trusting relationships and that patient partnerships must consider how to involve diverse communities so that health inequities are not reinforced.

### Challenge visible power to open up institutions to communities

As researchers, it is helpful to think through what resources, power and agency you might have access to, to establish a new culture of fostering trusting partnerships with communities and effecting change within systemic and institutional barriers that may exist. The next example further explores this, outlining how a group of researchers and civil servants were asked to develop a patient and public engagement strategy (Case study 5). Their instructions did not include any mention of, or resources to work with patients and users to develop this. The group recognised this omission, pushed back by demanding the mandate to conduct the work according to the group’ stipulated standards (such as including patients and users in this work) and thus changed the culture to co-develop the strategy with patient contributors.

Case study 5. Government Healthcare Agencies Collaboration with patients and public

**Table:** 

*What happened* In Sweden, the Council for Knowledge-Based Policy brings together all nine health and social care governmental agencies to support integration and collaboration. In 2016, the Council strengthened patient and user involvement across all nine agencies. Their initial plan was to form a working-group of agency members led by the S*tatens beredning för medicinsk och social utvärdering researchers* (*SBU)* (translation: Swedish Agency for Health Technology Assessment and Assessment of Social Services) to consider whether a common policy for patient and user involvement would be useful. All nine agencies volunteered representatives to participate in the working group for the first time. At the first meeting the working-group decided that in order to produce a meaningful policy of patient and user involvement, this needed to be co-developed with patients and users. *Consequences* A parallel patient and user group with representatives from various patient and user organisations was established. The leadership and meetings (space, expenses, refreshments) of this group was funded, but not the time of the members. The two groups decided together to mirror each other’s work, for example, when the working-group collected stories of successful collaborative examples, so did the patient and user group. Both groups had a participant in the other group and mutual meetings were also held. The project lasted one year, resulting in a common Council policy for patient and user involvement [31]. Both the working-group and patient and user group proposed this policy to all nine General Directors of the Council resulting in it being adopted across all nine agencies, which is an example of influencing visible power. *Lessons* When the Council was evaluated [32], both the example of the parallel working-group and patient and user group, and the policy was reviewed as one of only two promising initiatives. Over time the parallel working-group and patient and user group established a trusting relationship in which they could share different governmental and patient organisational agency institutional working practices and build solidarity to challenge each of the nine agencies to adopt the policy.

## Conclusion

Partnership approaches to research involve the public, researchers and professionals working together, sharing power in decision-making. Partnership approaches not only have the potential to improve the relevance and quality of the research and so ensure better research and health outcomes but also that people should be involved in decisions that impact upon them [1]. This is, nevertheless, a paradigm shift from more traditional ways of undertaking research and consulting people at various phases of the research cycle. Successfully collaborating involves considering the sources of power differentials (the visible, hidden and invisible), alongside the development of trust and relationships.

This paper has outlined three calls to actions, with examples from practice, in which researchers working in primary care can move towards more partnership approaches to research by reflecting on and challenging different forms of power. First, we suggest navigating invisible power dynamics within traditional research approaches by using creative and flexible more inclusive participatory methods. Second, we suggest tackling hidden power by embracing participatory approaches and including patients and the public in decision-making about the design, delivery and development of research and its outcomes. Third, we suggest challenging various systemic and institutional barriers which may hinder participatory approaches. If we can all commit to taking on some of these calls to action and consider what we can change within our spheres of influence, we can start to see how primary care research can be more welcoming and inclusive to all.
